# Leukocyte Ratios Associate With Vasodilatory Hemodynamics in Cardiac Intensive Care Shock

**DOI:** 10.1016/j.jacadv.2026.102684

**Published:** 2026-03-26

**Authors:** Patrick R. Lawler, Yishay Szekely, Lucas Lage Marinho, Dustin Hillerson, Jacob C. Jentzer

**Affiliations:** a Division of Cardiology and Interdepartmental Division of Critical Care Medicine, University Health Network, University of Toronto, Toronto, Canada; b Department of Medicine, McGill University Health Centre, McGill University, Montreal, Canada; c Department of Cardiology, Tel Aviv Sourasky Medical Center, Tel Aviv University School of Medicine, Tel Aviv, Israel; d Department of Cardiovascular Medicine, Mayo Clinic, Rochester, USA; e Robert D. and Patricia E. Kern Center for the Science of Health Care Delivery, Mayo Clinic, Rochester, USA

**Keywords:** biomarkers, cardiogenic shock, hemodynamics, inflammation, shock

**What is the clinical question being addressed?**

Do immune cell biomarkers track with shock profiles in the CICU?

**What is the main finding?**

The neutrophil:lymphocyte ratio tracked with vasodilatory hemodynamic profiles.

The syndrome of shock is comprised of hypoperfusion and organ dysfunction—resulting from low cardiac output or systemic vascular resistance (SVR), or a combination of both. Although shock is conventionally classified as either cardiogenic (low cardiac output) or vasoplegic (low SVR), many patients’ hemodynamic profiles manifest along a continuum between these entities, complicating shock management in the cardiac intensive care unit (CICU). Improved clinical tools to characterize shock etiology could support clinical care by providing insights into the markers and potential mediators of the hemodynamic course of shock in the CICU.

Systemic inflammation is variably activated by many of the events which incite shock, and by organ dysfunction itself; this may contribute to variable hemodynamic profiles in shock.^1^ The peripheral blood neutrophil:lymphocyte ratio (NLR)—derived from the standard clinical complete blood count—reflects the balance of innate and adaptive immune cell abundances.^2^^,^^3^ Although the NLR has been associated with early mortality in shock,^4^^,^^5^ its hemodynamic correlates remain uncharacterized. Identifying biomarkers of molecular and hemodynamic shock mechanisms may improve bedside clinical differentiation of shock etiologies and management in the CICU. We hypothesized that higher NLR may identify a subset of CICU patients with shock who have vasodilatory hemodynamic features.

## Methods

We performed a retrospective, single-center observational cohort study at the Mayo Clinic CICU, including consecutive adult patients (≥18 years) admitted with shock between 2007 and 2018 with an NLR and a comprehensive transthoracic echocardiogram within 24 hours of ICU admission. An admission diagnosis of shock was defined as an International Classification of Diseases (ICD)-9/10 diagnosis for shock documented by the CICU team, reflecting real-world practice across the study period. NLR was derived from the first complete blood count white blood cell differential obtained after admission, and taken as the ratio of the absolutely neutrophil count to the absolute lymphocyte count. Noninvasive hemodynamics were estimated by transthoracic echocardiography. Echocardiography was performed within 24 hours of admission. Clinical and echocardiographic findings were stratified across NLR quartiles. NLR quartiles were empirically derived based on the distribution of values within the study cohort given lack of a standard reference range for this marker and so as to allow for nonlinear relationships. Missing data were handled using complete-case analyses for each variable. The study was approved by The Mayo Clinic Institutional Review Board. Statistical analyses were performed using JMP Statistical Software (version 16.0.0, SAS Institute).

## Results

Among 1,132 patients (mean age 67.8 years, 38.3% women), median NLR was 9.84 (IQR: 5.94-15.81) (Table 1). Patients with higher NLR had higher illness severity, more organ failure, and more frequent receipt of vasopressors and mechanical ventilation—although inotrope and coronary angioplasty use were similar. Sepsis diagnosis was more common among patients with higher NLR (35% vs 22%). Higher NLR was associated with vasodilatory hemodynamic features, including lower diastolic blood pressure and lower SVR and indexed systemic vascular resistance (SVRi) (median SVRi ranged from 27.6 to 23.3 WU·m^2^, *P* = 0.009). Patients with higher NLR also had signs of congestion with higher right atrial and right ventricular pressures. Heart rate and cardiac index were higher among patients with higher NLR. Measures of left and right ventricular systolic function were similar across NLR quartiles. Consistent with prior observations from a larger sample of this cohort,^4^ in-hospital mortality and CICU length of stay increased stepwise (from 19.1% to 36.0%, *P* < 0.001; 2.68-3.90, *P* = 0.013, respectively) across increasing NLR quartiles.

**Table 1:** Baseline Characteristics, Admission Data, Hospital Outcomes, and Hemodynamics According to NLR Quartiles

	All Patients (N = 1,132)	1st Quartile (n = 283)	2nd Quartile (n = 282)	3rd Quartile (n = 284)	4th Quartile (n = 283)	*P* Value
Baseline data						
Demographics and baseline variables						
NLR^a^	9.84 (5.9-15.8)	4.17 (3.1-5.1)	7.67 (6.94-8.8)	12.2 (10.8-13.7)	23.25 (18.4-29.8)	<0.001
Age (y)	67.9 ± 13.8	66.0 ± 14.1	67.3 ± 13.1	68.3 ± 13.9	69.8 ± 13.9	0.001
Female	434 (38.3%)	112 (39.6%)	111 (39.4%)	101 (35.6%)	110 (38.9%)	0.65
Charlson Comorbidity Index	2.41 ± 2.51	2.40 ± 2.61	2.27 ± 2.46	2.22 ± 2.35	2.76 ± 2.58	0.13
SOFA score	5.62 ± 3.29	4.57 ± 3.43	5.86 ± 3.28	5.71 ± 3.14	6.33 ± 3.05	<0.001
Admission diagnosis^b^						
Cardiac arrest	371 (32.8%)	84 (29.7%)	85 (30.1%)	104 (36.6%)	98 (34.6%)	0.088
Cardiogenic shock	943 (83.3%)	245 (86.6%)	243 (86.2%)	234 (82.4%)	221 (78.1%)	0.003
Acute coronary syndrome	655 (57.9%)	171 (60.4%)	169 (59.9%)	165 (58.1%)	150 (53.0%)	0.067
Respiratory failure	755 (66.7%)	163 (57.6%)	186 (66.0%)	192 (67.6%)	214 (75.6%)	<0.001
Sepsis	294 (26.0%)	62 (21.9%)	69 (24.5%)	64 (22.5%)	99 (35.0%)	0.001
CICU procedures and therapies						
Invasive mechanical ventilation	643 (56.8%)	111 (39.2%)	175 (62.1%)	176 (62.0%)	181 (64.0%)	<0.001
Vasopressors	760 (67.1%)	155 (54.8%)	200 (70.9%)	202 (71.1%)	203 (71.7%)	<0.001
Inotropes	276 (24.4%)	77 (27.2%)	70 (24.8%)	60 (21.1%)	69 (24.4%)	0.29
PCI	397 (35.1%)	95 (33.6%)	110 (39.0%)	104 (36.6%)	88 (31.1%)	0.44
Intra-aortic balloon pump	390 (34.5%)	113 (39.9%)	102 (36.2%)	99 (34.9%)	76 (26.9%)	0.014
Intravenous antibiotics	598 (52.8%)	108 (38.2%)	156 (55.3%)	157 (55.3%)	177 (62.5%)	<0.001
Hemodynamics^c^						
Heart rate (beats/min)	86.4 ± 20.7	82.4 ± 21.0	88.0 ± 20.3	87.4 ± 21.3	87.9 ± 19.6	0.005
Systolic blood pressure (mm Hg)	107.5 ± 20.9	108.2 ± 21.8	106.9 ± 20.2	106.8 ± 20.9	108.1 ± 20.8	0.96
Diastolic blood pressure (mm Hg)	61.1 ± 14.4	63.1 ± 14.5	60.4 ± 14.2	60.7 ± 14.8	60.0 ± 13.7	0.021
Mean arterial pressure (mm Hg)	76.5 ± 14.5	78.1 ± 15.1	75.9 ± 14.0	76.1 ± 14.9	76.0 ± 13.9	0.12
Ventricular function^d^						
LVEF (%)	35 (25-52)	36 (23-51)	35 (22-50)	35 (23-51)	38.5 (25-56)	0.17
TAPSE (mm)	16 (12-19)	16 (14-20)	15 (12-19)	16 (12-19)	15 (12-19)	0.12
Cardiac output and systemic vascular resistance						
Cardiac index (L/min/m^2^)	2.66 ± 0.84	2.56 ± 0.79	2.64 ± 0.80	2.68 ± 0.90	2.76 ± 0.86	0.018
Systemic vascular resistance (WU)	12.6 (10.0-16.4)	14.2 (10.9-17.4)	12.9 (10.3-16.0)	12.2 (9.5-15.7)	11.2 (9.1-16.2)	0.008
Cardiac filling pressures						
RA pressure (mm Hg)	14 (10-20)	10 (5-16.5)	10 (10-15)	14 (10-20)	14 (10-20)	<0.001
RVSP (mm Hg)	45.7 ± 14.5	45.1 ± 15.3	44.5 ± 14.2	44.8 ± 13.8	48.2 ± 14.6	0.040
MV E/e’	15 (11.5-20)	15 (11.3-20)	15.7 (11.6-21.8)	15 (11.5-22.3)	16.3 (11.7-21.8)	0.45
Clinical outcomes						
CICU length of stay (days)	3.25 (1.8-5.82)	2.68 (1.43-4.83)	3.22 (1.65-5.79)	3.47 (1.94-6)	3.9 (2.09-6.69)	0.013
In-hospital mortality	310 (27.4%)	54 (19.1%)	74 (26.2%)	80 (28.2%)	102 (36.0%)	<0.001

Values are proportions (number [%]), mean (SD) for continuous traits, and median (IQR). Continuous variables were compared using analysis of variance or Kruskal-Wallis tests, as appropriate, and categorical variables using chi-square tests. Patients without available NLR or echocardiographic data within 24 hours of CICU admission were excluded from analysis. (A total of 1859 patients had shock, of whom 1,299 had a qualifying echocardiogram, of whom 1,132 has a qualifying NLR.).

CICU = cardiac intensive care unit; LVEF = left ventricular ejection fraction; MV = mitral valve; NLR = neutrophil:lymphocyte ratio; PCI = percutaneous coronary intervention; RA = right atrial; RVSP = right ventricular systolic pressure; SOFA = sequential organ failure assessment; TAPSE = tricuspid annular plane systolic excursion.

a Higher NLR was associated the combination of relative neutrophilia and relative lymphopenia. Median (IQR) absolute neutrophil counts increased steadily across NLR quartiles from 6.7 (4.9-8.9) to 14.2 (10.8-19.0) (*P* < 0.001), whereas absolute lymphocyte counts declined steadily across NLR quartiles from 1.7 (1.3-2.3) to 0.55 (0.4-0.6) (*P* < 0.001).

b Admission diagnoses were not mutually exclusive and may sum to >100%.

c Missing data excluded.

d Results were consistent for fractional shortening, myocardial contraction fraction index, and RV S’.

## Interpretation

In this large, single-center observational cohort study, higher peripheral blood NLR was more commonly associated with a vasodilatory hemodynamic features among CICU patients with shock. Elevated NLR was associated with lower SVR, compensatory tachycardia, organ dysfunction, and excess mortality, linking systemic innate immune activation to vasodilatory physiology. Collectively, these patterns track a vasodilatory phenotype superimposed on congestion—consistent with mixed cardiogenic-vasodilatory features. The NLR may be a clinically available, inexpensive biomarker of these processes.

Translationally, these findings may support the significance of immune activation in CICU shock. Clinically, high NLR at admission may identify patients in whom higher innate inflammatory activation promotes vasodilatory hemodynamic features, possibly identifying patients who potentially may derive differential benefit from mechanical circulatory support, inotropes, or vasopressors support—although further study is needed. NLR is an inexpensive, readily available laboratory marker.

Characterizing shock phenotypes in the CICU remains a major clinical challenge. Clinicians must synthesize a complex and often discordant set of data—including bedside assessments, invasive hemodynamics (eg, Swan–Ganz catheterization), echocardiography, and biomarker profiles—to classify shock and guide individualized management. Yet, accurate phenotyping frequently remains imprecise, and the natural history of shock phenotypes can be highly dynamic. The findings herein highlight the possibility for emerging biomarkers to refine the delineation of shock mechanisms in the CICU. Future work is needed to validate these and other candidate markers, and to define their incremental predictive value in other large well-phenotyped CICU shock cohorts.

This study has potential limitations. First, the presence and subtype of shock were defined using ICD-9/10 diagnosis codes documented by the treating team; definitions of shock evolved over the study period and standardized frameworks such as Society for Cardiovascular Angiography & Interventions staging were not uniformly available. As with shock, sepsis was defined using ICD codes rather than the study-standardized criteria. Given the observational design and collinearity between infection, systemic inflammation, and illness severity, sepsis may plausibly contribute to NLR-associated vasodilatory features, and causality cannot be inferred. SVR and SVRi were estimated noninvasively by echocardiography and may be imprecise in critically ill patients receiving vasoactive therapies; invasive hemodynamic validation was not available. In addition, the study is limited by lack of information on why an echocardiogram was or was not performed, lack of correlated Swan-Ganz catheter data, single center design, and absent microbiological data to complement available data on sepsis diagnoses and antibiotic use. Nevertheless, results were consistent across a range of hemodynamic measures. Future studies integrating detailed immune profiling with invasive monitoring could refine mechanistic endotypes and enable precision hemodynamic therapy in cardiogenic shock.

## Funding support and author disclosures

The study was funded by Kern Center for the Science of Health Care Delivery, Mayo Clinic, Rochester, USA. All other authors have reported that they have no relationships relevant to the contents of this paper to disclose.
